# Influence of *CYP2C9* Genetic Polymorphisms on the Pharmacokinetics of Losartan and Its Active Metabolite E-3174: A Systematic Review and Meta-Analysis

**DOI:** 10.3390/jpm11070617

**Published:** 2021-06-29

**Authors:** Yoon-A Park, Yu-bin Song, Jeong Yee, Ha-Young Yoon, Hye-Sun Gwak

**Affiliations:** College of Pharmacy and Graduate School of Pharmaceutical Sciences, Ewha Womans University, Seoul 03760, Korea; parkna123@ewhain.net (Y.-A.P.); 1564069@ewhain.net (Y.-b.S.); jjjhello1@naver.com (J.Y.); hayoungdymphnayoon@gmail.com (H.-Y.Y.)

**Keywords:** losartan, E-3174, *CYP2C9*, polymorphism, pharmacokinetics

## Abstract

This study aimed to investigate the influence of *CYP2C9* genetic polymorphisms on the pharmacokinetics of losartan and its active metabolite, E-3174, through a systematic review and meta-analysis. Eight studies published before March 2021 were included in this study. We used PubMed, the Cochrane Library, EMBASE, and Web of Science, based on the Preferred Reporting Items for Systematic Reviews and Meta-Analyses (PRISMA) guidelines. The data analysis was conducted through Review Manager (RevMan), version 5.3, and R software. We found that healthy volunteers with CYP2C9*2 or *3 carriers had higher area under the curve (AUC_0-∞_) of losartan (mean difference (MD) 0.17 μg·h/mL; 95% confidence intervals (CI): 0.04, 0.29) and lower AUC_0-∞_ of E-3174 (MD −0.35 μg·h/mL; 95% CI: −0.62, −0.08) than those with CYP2C9*1/*1. Subjects with CYP2C9*2 or *3 carriers showed lower maximum concentration (C_max_) of E-3174 than those with CYP2C9*1/*1 (MD −0.13 μg/mL; 95% CI: −0.17, −0.09). For half-life, subjects with CYP2C9*2 or *3 carriers had longer half-lives of losartan and E-3174 than those with CYP2C9*1/*1 (MD 0.47 h; 95% CI: 0.32, 0.61 and MD 0.68 h; 95% CI: 0.44, 0.92, respectively). This meta-analysis suggests that the pharmacokinetics of losartan and E-3174 are associated with the *CYP2C9* polymorphisms

## 1. Introduction

Losartan is an angiotensin II receptor blocker (ARB) that is widely used for hypertension, heart failure, and diabetic nephropathy. It blocks the angiotensin II type 1 (AT1) receptor. It is absorbed from the gastrointestinal tract after oral administration and undergoes substantial first-pass metabolism, resulting in a systematic bioavailability of approximately 33%. It is metabolized to an active carboxylic acid metabolite E-3174, which has up to 40 times greater pharmacological activity than losartan [1,2].

Cytochrome P450 (CYP) 2C9 comprises approximately 20% of CYP enzymes in the human liver, where it metabolizes more than 100 clinical drugs, including losartan [3]. CYP2C9 metabolizes losartan to E-3174 by oxidation of the C5-hydroxymethyl on the imidazole ring of the 5-carboxylic acid. *CYP2C9* is highly polymorphic, with at least 30 different variants. Among them, CYP2C9*2 (430T > C, Arg144Cys) and CYP2C9*3 (1075A > C, Ile359Leu) are the two most well-studied alleles. These alleles reportedly decrease the activity of CYP2C9 [3].

As the *CYP2C9* gene plays an important role in losartan pharmacokinetics, there are several studies on the effects of *CYP2C9* polymorphisms on losartan pharmacokinetic parameters, such as the area under the curve (AUC_0-∞_), maximum concentration (C_max_), and half-life. Individuals with CYP2C9*2 or *3 alleles reportedly undergo poorer metabolism than those with CYP2C9*1/*1 [4]. CYP2C9*2 or *3 carriers have a higher ratio of plasma AUC_0-∞_ of losartan to AUC_0-∞_ of E-3174 than those with CYP2C9*1/*1 [3]. In contrast, Burnier et al. reported that the AUC_0-∞_ of E-3174 in individuals with CYP2C9*1/*3 was not significantly lower than those with CYP2C9*1/*1 [5].

Safe and effective drug therapy requires an understanding of a drug’s pharmacokinetic, pharmacodynamic, and pharmacogenomic interrelationships [6]. Drug response and adverse events can be predicted using pharmacokinetic parameters [7]. Genetic polymorphisms can affect drug concentrations and effectiveness. However, the association between *CYP2C9* gene polymorphisms and losartan pharmacokinetic parameters has been inconsistent, possibly due to small sample sizes [8,9,10,11]. This study aimed to investigate the effects of the *CYP2C9* polymorphisms on the pharmacokinetic characteristics of losartan and E-3174 through systematic review and meta-analysis.

## 2. Methods

The paper was conducted based on the Preferred Reporting Items for Systematic Reviews and Meta-Analyses (PRISMA) guidelines [12].

### 2.1. Search Strategy

Two investigators independently conducted a systemic search for all studies published before 4 March 2021, using PubMed, Cochrane Library, EMBASE, and Web of Science. The following search terms were included: (losartan OR (losartan potassium) OR Cozaar) AND ((CYP2C9*) OR (cytochrome p450 2C9) OR (cytochrome p 450 2C9)) AND (polymorph* OR variant* OR mutation* OR genotyp* OR phenotyp* OR haplotyp* OR allele* OR SNP* OR pharmacogen*).

### 2.2. Selection Criteria and Data Extraction

Studies were selected if they (1) compared the pharmacokinetic characteristics of subjects with CYP2C9*2 or *3 alleles to those with CYP2C9*1/*1 after oral administration of losartan; (2) included healthy adults who received a single dose of 50 mg losartan; and (3) were randomized, controlled trials (RCT) or cohort studies.

Studies were excluded if they (1) were editorials, notes, abstracts, reviews, news, letters, posters, or comments; (2) were in vitro or animal studies; (3) did not include blood sample data; (4) had concomitant medications with losartan; or (5) were unable to extract outcome data. If there were overlapping data, only the most recent and comprehensive data were included in the meta-analysis.

The following parameters were extracted independently by two investigators: name of the first author, year of publication, nation, studied polymorphisms, age, number of subjects, body mass index (BMI), genotyping methods, and quantitative methods. The AUC_0-∞_ (primary outcome), C_max_, and half-life (secondary outcomes) of losartan and E-3174 were also extracted from each study.

Study quality was assessed by the Newcastle-Ottawa scale (NOS) tool [13]. The NOS tool assessment is based on three primary domains, including selection of subjects (0–4 points), comparability of study groups (0–2 points), and determination of outcomes of interest (0–3 points).

### 2.3. Statistical Analysis

The mean difference (MD) with 95% confidence intervals (CIs) was calculated to compare AUC_0-∞_, C_max_, and half-life. CYP2C9*2 or *3 carriers were compared with CYP2C9*1/*1. We also compared the two groups (CYP2C9*3 carriers and CYP2C9*1/*1). Heterogeneity was evaluated by Cochrane’s Q statistic and Higgins’ I^2^ statistics [14]. The random-effects model was applied when heterogeneity existed (*I*^2^ > 50%); otherwise, the fixed-effects model was applied.

We performed a subgroup analysis by ethnicity and conducted a sensitivity analysis, using sequential omission of each study, to validate the robustness of the results. Begg’s rank correlation test and Egger’s regression test were used to detect publication bias. Statistical analyses were performed using Review Manager (RevMan) version 5.3 (The Cochrane Collaboration, Copenhagen, Denmark) and R software (version 4.0.5; R Foundation for Statistical Computing, Vienna, Austria). A *p*-value < 0.05 was considered statistically significant.

## 3. Results

**Figure 1:**
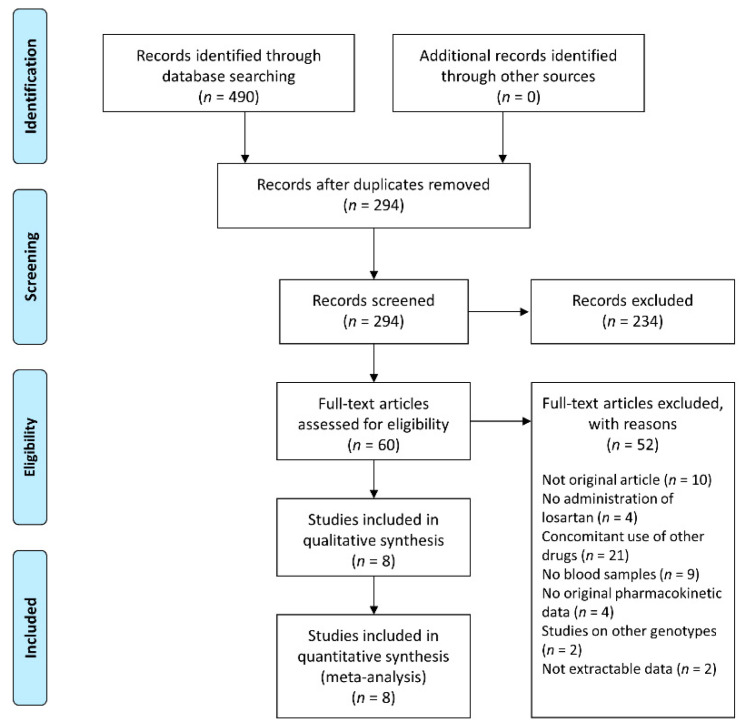
Flow diagram of study selection.

The literature search resulted in 490 articles, 294 of which remained after duplicates were removed, and 234 of which were excluded based on the title and abstract. We excluded 52 articles for the following reasons: (1) not original articles (*n* = 10); (2) no losartan administration (*n* = 4); (3) subjects administered other drugs concomitantly (*n* = 21); (4) no blood sample data (*n* = 9); (5) no original pharmacokinetic data (*n* = 4); (6) studies on other genotypes (*n* = 2); and (7) not extractable data (*n* = 2). Eight articles remained after assessing full-text articles (Figure 1). The characteristics of these studies are presented in Table 1 [8,15,16,17,18,19,20,21]. Five studies were published in Asia, two studies were conducted in Europe, and one study was in the United States. The studies were published between 2002 and 2021. The NOS ranged from 6 to 7 (Table 1).

**Figure 2:**
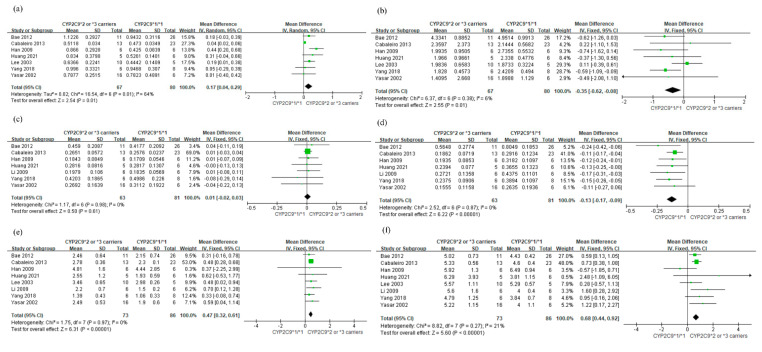
Forest plot comparing the mean difference between CYP2C9*2 or *3 carriers and CYP2C9*1/*1. (**a**) AUC_0-∞_ of losartan (μg·h/mL), (**b**) AUC_0-∞_ of E-3174 (μg·h/mL), (**c**) C_max_ of losartan (μg/mL), (**d**) C_max_ of E-3174 (μg/mL), (**e**) half-life of losartan (h), (**f**) half-life of E-3174 (h).

Based on the seven studies in Figure 2, subjects with CYP2C9*2 or *3 carriers showed higher AUC_0-∞_ of losartan than those with CYP2C9*1/*1 (MD 0.17 μg·h/mL; 95% CI: 0.04, 0.29) (Figure 2a). Heterogeneity was detected among the studies ($I^{2\;}$ = 64%; *p* = 0.01). In contrast, subjects with CYP2C9*2 or *3 carriers showed significantly lower AUC_0-∞_ of E-3174 compared to those with CYP2C9*1/*1 (MD −0.35 μg·h/mL; 95% CI: −0.62, −0.08), with low heterogeneity ($I^{2}$ = 6%) (Figure 2b).

For C_max_ of losartan, there was no significant difference between subjects with CYP2C9*2 or *3 alleles and those with CYP2C9*1/*1 (MD 0.01 μg/ mL; 95% CI: −0.02, 0.03) (Figure 2c). Heterogeneity was not detected among the studies ($I^{2}$ = 0%). However, subjects with CYP2C9*2 or *3 variants showed lower C_max_ of E-3174 than those with CYP2C9*1/*1 (MD −0.13 μg/mL; 95% CI: −0.17, −0.09) (Figure 2d), with no heterogeneity ($I^{2}\;$= 0%). Subjects with the CYP2C9*2 or *3 carriers showed significantly longer half-lives of losartan and E-3174 than those with the CYP2C9*1 carrier (MD 0.47 h; 95% CI: 0.32, 0.61 and MD 0.68 h; 95% CI: 0.44, 0.92, respectively) (Figure 2e,f). Neither Begg’s test nor Egger’s test showed significant publication bias for all analyses.

**Figure 3:**
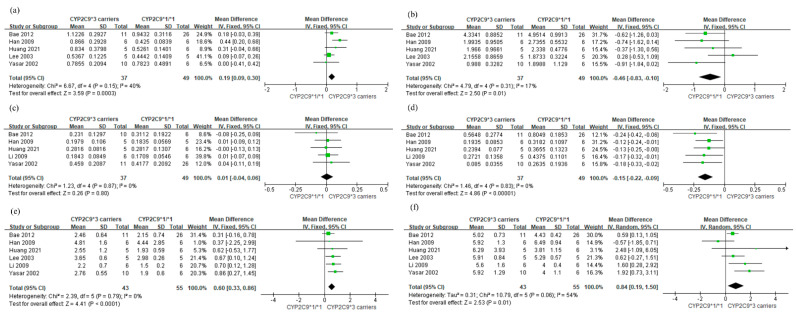
Forest plot comparing the mean difference between CYP2C9*3 carriers and CYP2C9*1/*1. (**a**) AUC_0-∞_ of losartan (μg·h/mL), (**b**) AUC_0-∞_ of E-3174 (μg·h/mL), (**c**) C_max_ of losartan (μg/mL), (**d**) C_max_ of E-3174 (μg/mL), (**e**) half-life of losartan (h), (**f**) half-life of E-3174 (h).

**Figure 4:**
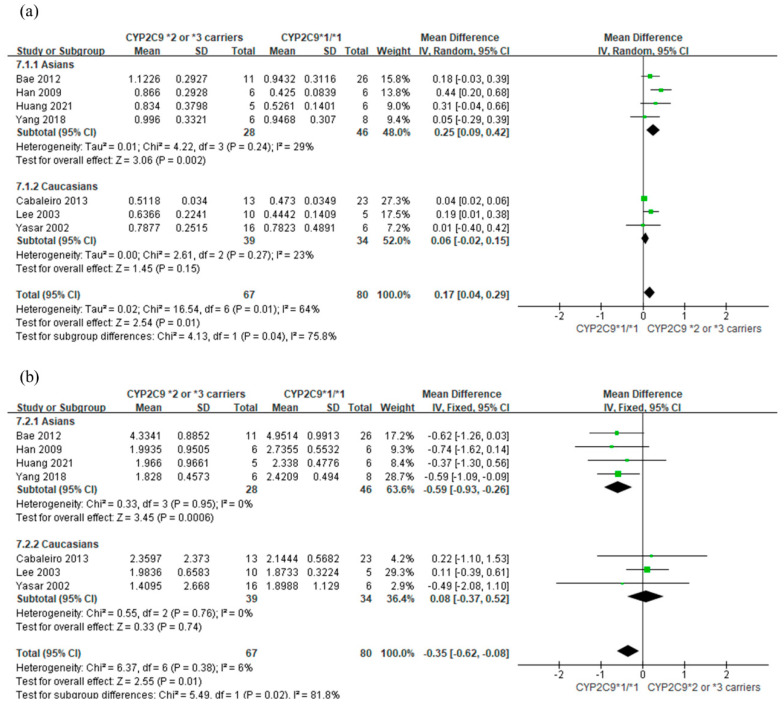
Forest plot with subgroups of Asians and Caucasians for comparing the mean difference between CYP2C9*2 or *3 carriers and CYP2C9*1/*1. (**a**) AUC_0-∞_ (μg·h/mL) of losartan and (**b**) AUC_0-∞_ (μg·h/mL) of E-3174.

In a comparison between CYP2C9*3 carriers and CYP2C9*1/*1 (Figure 3), the analysis results were similar to that between CYP2C9*2 or *3* carriers and CYP2C9*1/*1 (Figure 2). Additionally, a subgroup analysis was conducted by ethnicity (Figure 4). There was a significant difference in the AUC_0-∞_ of losartan (*p* = 0.04) and AUC_0-∞_ of E-3174 (*p* = 0.02) depending on the ethnic subgroup. In the Asian subgroup, subjects with CYP2C9*2 or *3 carriers had a higher AUC_0-∞_ of losartan than those with CYP2C9*1/*1 (MD 0.25 μg/mL; 95% CI: 0.09, 0.42). In contrast, the AUC_0-∞_ of losartan was not significantly different between those with CYP2C9*2 or *3 alleles and with CYP2C9*1/*1 in the Caucasian subgroup (MD 0.06 μg/mL; 95% CI −0.02, 0.15). The MD of AUC_0-∞_ of E-3174 between CYP2C9*2 or *3 genotypes and CYP2C9*1/*1 for Asians and Caucasians were −0.59 (95% CI −0.93, −0.26) and 0.08 (95% CI −0.37, 0.52), respectively.

### Sensitivity Analysis

To assess the stability of the results, a sensitivity analysis was performed by sequentially excluding each study. Sensitivity analysis of AUC_0-∞_ of losartan and E-3174 showed similar results to the main analysis; MD between CYP2C9*2 or *3 carriers and CYP2C9*1/*1 ranged from 0.09 to 0.22 for AUC_0-∞_ of losartan and −0.54 to −0.25 for AUC_0-∞_ of E-3174, respectively. When the study by Cabaleiro et al. was eliminated, the heterogeneity of losartan AUC_0-∞_ decreased from 64% to 11% [16].

## 4. Discussion

This is the first meta-analysis to evaluate the effect of *CYP2C9* gene polymorphisms on the pharmacokinetic properties of losartan. The results showed that CYP2C9*2 or *3 carriers had a higher AUC_0-∞_ of losartan and lower AUC_0-∞_ of E-3174 than those with CYP2C9*1/*1. There was no significant difference in the C_max_ of losartan between subjects with CYP2C9*2 or *3 and CYP2C9*1/*1, whereas the subjects with CYP2C9*2 or *3 carriers had lower C_max_ of E-3174 than those with CYP2C9*1/*1. The half-lives of both losartan and E-3174 were longer in CYP2C9*2 or *3 carriers than CYP2C9*1/*1. Similar results were obtained in the comparison between CYP2C9*1/*1 and CYP2C9*3 carriers. There was an ethnic difference in the effects of *CYP2C9* gene polymorphisms on the AUC_0-∞_ values of losartan and E-3174 between Asians and Caucasians.

There are several studies on the effect of CYP2C9 inhibitors, such as fluconazole, bucolome, and ticlopidine, on the pharmacokinetic parameters of losartan. An in vitro study revealed that the C_max_ and AUC_0-∞_ of E-3174 significantly decreased to 30% and 47% by fluconazole, respectively, compared to the control group [22]. Kobayashi et al. reported that concomitant use of losartan and bucolome resulted in the significant decrease of AUC_0-∞_ of E-3174 and increase of AUC_0-∞_ of losartan in healthy male volunteers [23]. There was also a report that ticlopidine increased the AUC_0-∞_ of losartan in rats [24]. The aforementioned studies indicate that the CYP2C9 enzyme was a significant metabolizer of losartan.

The pharmacokinetic properties of losartan can affect its response. One study found that the CYP2C9*3 variant reduces losartan metabolism and its hypotensive effect after oral administration of losartan [25]. In this study, the C_max_ of E-3174 in the CYP2C9*1/*3 group was 30% lower than that in the CYP2C9*1/*1 group. Subjects with CYP2C9*1/*3 had a less hypotensive effect in the diastolic blood pressure (DBP) at 10 h and 12 h post-dosing than those with CYP2C9*1/*1. The CYP2C9*1/*1 group had a more significant decrease in the systolic blood pressure (SBP) from 1 h to 12 h than the CYP2C9*1/*3 group.

*CYP2C9* polymorphism may differently affect the ARB drug responses. Losartan has lower efficiency in CYP2C9*2 or *3 carriers, despite an increased concentration of losartan. The low efficiency in CYP2C9*2 or *3 carriers was attributed to the decreased AUC_0-∞_ of E-3174, which has more potent activity than losartan. In contrast, in the case of irbesartan, the CYP2C9*1/*3 genotype carriers showed both a higher concentration of irbesartan and greater DBP responses compared to the CYP2C9*1/*1 genotype carriers; this was possibly because the metabolite has no pharmacological activity [26]. Chen et al. also showed that subjects with the CYP2C9*1/*3 genotype had significantly higher plasma irbesartan concentrations compared with those with the CYP2C9*1/*1 genotype [27].

We further investigated the effect of the complex heterozygous genotype of CYP2C9*2 and CYP2C9*3 on losartan pharmacokinetics or pharmacodynamics. There was only one study to examine the pharmacokinetic difference between patients with both CYP2C9*2 and CYP2C9*3 alleles and those with CYP2C9*1/*1 [11]. Subjects with the complex heterozygous genotype of CYP2C9*2 and CYP2C9*3 had a significantly higher C_max_ and AUC of E-3174 than those with CYP2C9*1/*1 (179 vs. 603 nmol/L and 2134 vs. 4346 nmol/L∙h, respectively). However, in the case of losartan, the C_max_ and AUC did not show a statistically significant difference between CYP2C9*2/*3 and CYP2C9*1/*1 (635 vs. 675 nmol/L and 1697 vs. 2006 nmol/L∙h, respectively).

CYP2C9*2 and *3 frequencies are higher in Caucasians than in Asians [4,28,29]. In the *CYP2C9* polymorphisms in Asians, the CYP2C9*3 frequencies are more dominant than CYP2C9*2 (3.55% vs. 0.25%) [29]. Among Caucasians, the CYP2C9*2 allele is more frequent than the CYP2C9*3 allele (8.0% vs. 6.0%) [4]. Overall, in this study, a higher MD of the AUC_0-∞_ of losartan was observed in the Asian subgroup than in the Caucasian subgroup (0.25 μg·h/mL vs. 0.06 μg·h/mL). The *CYP2C9* polymorphisms in Asians were mostly CYP2C9*1/*3 in this study. On the contrary, in the Caucasian subgroup, the CYP2C9*2 carriers, such as CYP2C9*1/*2 and CYP2C9*2/*2, were more common than in the Asian subgroup. The ethnic difference in the MD by *CYP2C9* polymorphisms is possibly due to different distributions of *2 and *3, considering that the CYP2C9*3 allele has lower enzyme activity than the CYP2C9*2 allele [30].

Polymorphisms of CYP2C9 should be considered in the case of antihypertensive drug polytherapy, because other hypertensive agents, such as carvedilol, torsemide, and indapamide, are also known to be CYP2C9 substrates [31]. According to Pan et al., *CYP2C9* variants decreased the intrinsic clearance of carvedilol [32]. For torsemide, it was shown that CYP2C9*3 resulted in lower oral clearance and metabolite formation clearance [33]. In the study of Wang et al., patients with homozygous variants of *CYP2C9* rs4918758, which showed lowered CYP2C9 activity, had higher C_max_ and AUC and lower clearance of indapamide [34]. As CYP2C9 is involved in the metabolism of several antihypertensive agents, *CYP2C9* loss-of-function alleles may increase the parent drug level. There are some limitations to this meta-analysis that should be considered when interpreting the results. First, the limited number of studies may lead to low statistical power in detecting differences or heterogeneity. However, according to Herbison et al., meta-analyses with as few as four or five studies could produce robust results that are consistent with long-term results [35]. Second, some potential confounder variables, which could be associated with pharmacokinetics (e.g., kidney and liver functions), could not be adjusted due to a lack of information from individual studies. Third, only healthy volunteers were involved in this study, indicating that the results may not be applicable to patients. Fourth, other *CYP2C9* genotypes, such as CYP2C9*13, were not included in this meta-analysis because of low frequencies. Fifth, we could not conduct a meta-analysis comparing CYP2C9*2 carriers with CYP2C9*1/*1 carriers due to a lack of studies.

Despite these drawbacks, this study is the first systematic review and meta-analysis to evaluate the association between *CYP2C9* genotypes and pharmacokinetic characteristics of losartan and its active metabolite. By combining the results of several studies, this study suggests that CYP2C9*2 or *3 alleles may be significantly associated with the pharmacokinetics of losartan and its active metabolite.

In conclusion, we found that CYP2C9*2 or *3 carriers showed higher AUC_0-∞_ of losartan and lower AUC_0-∞_ of E-3174 compared to those with CYP2C9*1/*1. As altered pharmacokinetics can affect the therapeutic responses of losartan, genotyping CYP2C9 may be useful in understanding individual pharmacokinetic and pharmacodynamic differences.

## Figures and Tables

**Table 1:** Characteristics of studies included.

First Author, Year	Nation	Studied Polymorphisms	Age	n (Male Percent, %)	BMI (kg/m^2^) (SD)	Genotyping Methods	Quantitative Methods	Total NOS
Bae et al. 2012 [15]	Korea	CYP2C9*3	22.6 (1.5 ^b^)	13 (N/A)	22.6 (2.3^ b^)	PCR-RFLP	HPLC-FLU	7
Cabaleiro et al. 2013 [16]	Spain	CYP2C9*2 CYP2C9*3	22.6 (1.6^ b^)	36 (50.0)	72.1 ^c^ (6.2^ b,c^)	RT-PCR	HPLC-MS/MS	6
Han 2009 et al. [17]	China	CYP2C9*3	21.9 (2.6^ b^)	12 (100.0)	24.6 (4.9^ b^)	PCR-RFLP	HPLC-MS	7
Huang 2021 et al. [18]	China	CYP2C9*3	23.0 (N/A^ b^)	11 (N/A)	19.6 (N/A^ b^)	PCR-RFLP	HPLC-MS	7
Lee 2003 et al. [8]	United States	CYP2C9*2 CYP2C9*3	24.0 (5.0^ b^)	15 (47.0)	79.0 ^c^ (18.0^ b,c^)	N/A	HPLC-FLU	7
Li 2009 et al. [19]	China	CYP2C9*3	20.1 (1.9^ b^)	16 (100.0)	17.0–19.0 ^a^	PCR-RFLP	HPLC-MS/MS	6
Yang 2018 et al. [20]	China	CYP2C9*2	21–25 ^a^	14 (50.0)	17.4–24.8 ^a^	PCR-RFLP	HPLC-FLU	7
Yasar 2002 et al. [21]	Sweden	CYP2C9*2 CYP2C9*3	25–54 ^a^	22 (50.0)	52–91 ^a,c^	PCR-RFLP	HPLC-FLU	7

Index; BMI: body mass index; HPLC-FLU: high performance liquid chromatography with fluorescence; HPLC-MS/MS: high performance liquid chromatography with tandem mass spectrometry; N/A: Not Available; NOS: Newcastle-Ottawa score; PCR-RFLP: polymerase chain reaction–restriction fragment length polymorphism; RT-PCR: real time-polymerase chain reaction; SD: standard deviation. ^a^ range, ^b^ standard deviation, ^c^ weight (kg).

## Data Availability

No new data were created or analyzed in this study. Data sharing is not applicable to this article.
